# Costs of UK community care for individuals with recessive dystrophic epidermolysis bullosa: Findings of the Prospective Epidermolysis Bullosa Longitudinal Evaluation Study

**DOI:** 10.1002/ski2.314

**Published:** 2024-01-10

**Authors:** Eunice Jeffs, Elizabeth Pillay, Lesedi Ledwaba‐Chapman, Alessandra Bisquera, Susan Robertson, John McGrath, Yanzhong Wang, Anna Martinez, Anita Patel, Jemima Mellerio

**Affiliations:** ^1^ St John's Institute of Dermatology Guy's and St Thomas' Hospitals NHS Trust London UK; ^2^ Department of Population Health Sciences King's College London London UK; ^3^ Department of Dermatology The Royal Children's Hospital Parkville Victoria Australia; ^4^ Department of Dermatology Royal Melbourne Hospital Melbourne Victoria Australia; ^5^ Department of Dermatology King's College London Faculty of Life Sciences and Medicine London UK; ^6^ Department of Dermatology Great Ormond Street Hospital for Children London UK; ^7^ Anita Patel Health Economics Consulting Ltd London UK

## Abstract

**Background:**

Recessive dystrophic epidermolysis bullosa (RDEB) is a rare inherited skin fragility disorder requiring multidisciplinary management. Information regarding costs of current standard treatment is scant.

**Objectives:**

As part of a longitudinal natural history study, we explored the community care costs of UK patients with different forms of RDEB.

**Methods:**

Fifty‐nine individuals with RDEB provided detailed information on multiple facets of RDEB including disease severity scores (iscorEB, BEBS) and patient reported outcomes (quality of life evaluation in epidermolysis bullosa, iscorEB patient questionnaire). Costs data included time spent doing dressings, frequency of dressing changes, details of materials used, and paid and unpaid care.

**Results:**

Overall costs of dressing materials and associated care were high in RDEB. Median annual costs across all subtypes for those using dressings (*n* = 51) were over £26 000. For severe RDEB (RDEB‐S), median costs were almost £90 000 per annum, with a median of 18 h per week spent on dressing changes. Half of working‐age adults with RDEB were unemployed and 39% of carers were unable to take on full‐time or part‐time paid employment, adding to indirect costs and the financial burden from RDEB on families and society.

**Conclusions:**

The findings demonstrate the high costs of care of RDEB, particularly for RDEB‐S. The current expense supports the drive to develop new therapies which accelerate wound healing and diminish total wound burden, thereby reducing costs of dressings and care. While costly to bring to market, these might ultimately reduce the overall cost of treatment and also the impact on individuals living with this rare disease. The data also highlight the need for adequate reimbursement for EB care which can place significant financial strain on families.

## Graphical abstract

This article reports findings related to community care costs for individuals with different forms of recessive dystrophic epidermolysis bullosa (RDEB) from the Prospective Epidermolysis Bullosa Longitudinal Evaluation Study (PEBLES). It provides detailed information on the real‐world costs of community care including wound care materials used (dressings, topical treatments, retention garments, bandages), time spent doing dressings, frequency of dressing changes, and paid and unpaid care. It also explores correlations between the cost of community care and disease severity.

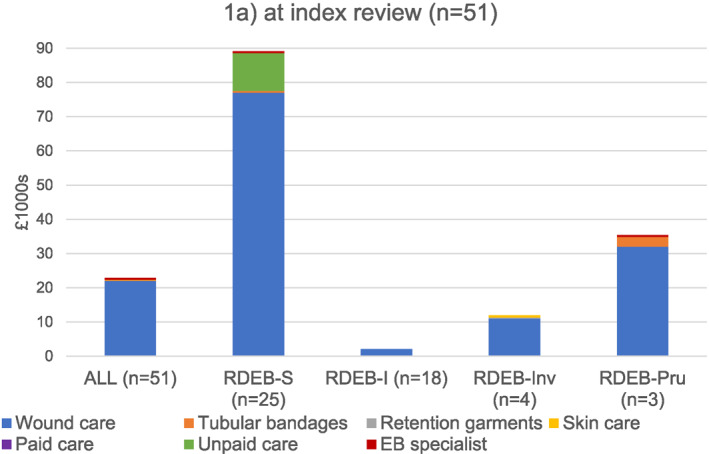

1

1 **What is already known about this topic?**

- Recessive dystrophic epidermolysis bullosa (RDEB) is a complex, multisystem disease requiring considerable hospital‐ and community‐based care throughout life.
- A small number of studies have explored costs incurred in different elements of Epidermolysis bullosa (EB) care but have relied largely on self‐reported measures, or incomplete health records or insurance data.

**What does this study add?**

- Our study provides detailed information obtained by participant interview and self‐report questionnaires regarding the real‐world costs of UK community care (including wound care materials) for individuals with different RDEB subtypes.
- In severe RDEB (RDEB‐S), individuals spend a median of 18 h per week on dressing changes, and total median annual community care costs almost £90 000.

## 2 INTRODUCTION

Epidermolysis bullosa comprises a group of rare inherited mucocutaneous fragility disorders. The overall prevalence per million population in the Netherlands^1^ and UK^2^ in 2020 was 22.4–34.8 compared with an estimated 11.1 prevalence in the USA in 2002.^3^ Reported UK prevalence of recessive dystrophic EB (RDEB) ranges from 1.4 to 3.3 per million, with incidence between 3.05 and 8.1 per million live births.^2^ Skin erosions and chronic wounds are common and may be extensive requiring expensive specialised dressings which can be time‐consuming to change. In RDEB, particularly the severe form, RDEB‐S, extracutaneous complications are frequent and lifespan can be significantly foreshortened,^4^ ^,^ ^5^ notably from aggressive cutaneous squamous cell carcinomas developing in early adult life.^6^ ^,^ ^7^ Management of EB is multidisciplinary and intensive, by necessity, associated with high healthcare and resource use costs,^8^ including direct medical costs (dressings, medications, hospital appointments, procedures), direct non‐medical costs (paid care), and indirect costs (productivity losses from patients and family members unable to undertake paid employment due to care needs).^9^

Rare or ‘orphan’ disorders such as EB previously attracted little attention from biopharmaceutical companies; developing and bringing new therapies to market was considered financially unviable while benefitting relatively few individuals. However, arguments for developing new therapies for rare diseases may be more complex than a straight cost/benefit analysis.^10^ Increased attention is focussing on the economics of delivering lifelong care with some governments and regulatory authorities offering financial frameworks to support development of new therapies.^11^, ^12^, ^13^, ^14^, ^15^

Costs of illness studies are important in defining healthcare and research priorities for governments and biopharmaceutical companies.^16^ However, such studies in rare diseases, including EB, are scant.^17^ ^,^ ^18^ A questionnaire‐based study in 8 European countries in 2016 reported average annual care costs of €31,390 for all EB types.^8^ Further analysis of the same data in 2022 revealed an average care cost of €53,359 for 91 individuals with DEB from 5 European countries, including the UK,^9^ although they did not stratify by DEB subtype or include costs of wound care materials. There is international consensus regarding EB care although resourcing of that care varies according to the healthcare funding system in individual nations. For example, UK healthcare is provided by the National Health Service (NHS), funded through general taxation and National Insurance contributions; patients do not directly pay for treatment except contributions (annual maximum of £112 (GBP, 2023)) towards prescription items such as wound dressings, medications, dental care. High‐cost rare diseases, including EB, are funded from ring‐fenced centralised NHS monies and provided by a limited number of designated Highly Specialised Services.

Ascertaining accurate data regarding full costs of care in EB is essential to ensure adequate budgeting and remuneration by national healthcare providers and/or insurers, and for biopharmaceutical companies and regulatory bodies when considering development of new therapies. Our study explores UK community care costs for different RDEB subtypes in significant depth, including the first detailed costs of wound care materials.

## 3 PATIENTS AND METHODS

### 3.1 Study population

The Prospective Epidermolysis Bullosa Longitudinal Evaluation Study (PEBLES) is an ongoing prospective register study recruiting individuals from the London EB centres, Great Ormond Street Hospital (children) and Guy's and St Thomas' Hospital (adults). Recessive dystrophic epidermolysis bullosa diagnosis was confirmed by skin biopsy and/or genetic testing with subtype determined by clinical features. Data are reported for participants recruited between 19^th^ November 2014 and 17^th^ November 2021. Reviews were undertaken 6‐monthly in under‐10s and annually for those 10 years and older, updating information since the preceding visit, including EB‐ and non‐EB‐related health issues, disease severity scores, subjective data including itch, pain and quality of life, results of laboratory and imaging studies, information relating to costs of care. Data were collected by a research nurse mostly through face‐to‐face interviews and self‐report questionnaires which were discussed with the participant; during the Covid‐19 pandemic the study team conducted virtual participant reviews (telephone or video call) and routine patient care was maintained by clinical teams at each site. Data were pseudonymised and recorded in a Research Electronic Data Capture database, retaining birthdate to link participants' age to reviews. Prospective Epidermolysis Bullosa Longitudinal Evaluation Study was ethically approved by the UK Research Ethics Committee and Health Research Authority (IRAS 142032).

### 3.2 Cost of care data

To capture ‘real world’ healthcare and resource use costs, participants and carers were asked about types and quantities of materials used during an average week, including wound care materials (dressings, topical treatments such as wound gel, reusable retention garments/tubular bandages), skin care and hygiene products (prescribed ointments, bath additives, moisturising creams), paid and informal (unpaid) carer time. These community care costs associated with delivering EB care at home are mostly funded by the NHS with some paid carers funded through social care provided by their local council (local government). In addition, the adult EB service provided clinical nurse specialist (EB‐CNS) home visits whereas children were typically seen in the hospital.

Community care costs are reported as British Pounds (GBP, £ or £1000s) per annum, with individual costs calculated using NHS unit costs for 2020^19^, ^20^, ^21^; preferred skin care items purchased by the participant, such as shower gel and moisturizers from the supermarket, were excluded from the analysis as not a cost to the NHS. All paid care was calculated at £12.50 per hour (average band 3 nurse NHS rate, 2020),^22^ with informal care for dressing changes valued as if provided by a professional carer.^23^ EB‐CNS costs were calculated by the hospital as £478 per visit.

### 3.3 Severity scores

Disease severity was scored using 2 validated questionnaires: instrument for scoring clinical outcomes of research for EB (iscorEB) (maximum clinician score of 138, self‐reported score of 120)^24^ and Birmingham EB Severity Score (BEBS) (maximum score of 100).^25^ Higher scores indicated greater RDEB activity/severity. We separately report skin wounding components as indicators of severity: BEBS reports percentage damaged skin including blisters, erosions, healing skin, erythema, atrophic scarring; iscorEB reports a composite score comprising intact skin, erosions, crusting/scabbing, chronic wounds (>6 weeks), infection and percentage body surface area affected. Financial impact of living with EB was assessed using quality of life evaluation in epidermolysis bullosa (QOLEB) questionnaire, item 3.^26^

### 3.4 Statistical analysis

To provide a snapshot of costs for all RDEB and by subtype, findings are presented for (1) the index visit (first available review with complete costs data) and (2) an average of per‐participant costs from all available reviews. Categorical variables are reported as counts and percentages, with continuous variables summarised using medians and inter‐quartile range [IQR]. Correlations were computed using Spearman's rank correlation. All analyses were performed using R (version 4.1.3).

Six participants had incomplete index reviews with missing iscorEB data: two children and one adult with RDEB‐S, three adults with intermediate RDEB (RDEB‐I). Also, one participant was missing the number of EB‐CNS visits. Data for the sole participant with pretibial RDEB (RDEB‐PT) (5 reviews) were included only in overall analysis. Missing‐ness of data are reported where relevant in the tables and figures.

## 4 RESULTS

Fifty‐nine participants provided complete costs data for a median 6 [4;7] reviews, totalling 330 reviews, including 25 individuals with RDEB‐S (10 recruited as children), 21 with RDEB‐I (4 recruited as children), 9 with inversa RDEB (RDEB‐Inv), 3 with RDEB pruriginosa (RDEB‐Pru), 1 with RDEB‐PT (Table 1). Individuals with RDEB‐S and RDEB‐Pru reported greater markers of severity than those with RDEB‐I and RDEB‐Inv, including iscorEB, BEBS and more frequent dressing changes (Table 1, index reviews; Table S1, all reviews).

**TABLE 1:** Participant characteristics by recessive dystrophic epidermolysis bullosa (RDEB) subtype at index review (*n* = 59).

Characteristic	Category	Overall	RDEB‐S	RDEB‐I	RDEB‐Inv	RDEB‐Pru	RDEB‐PT
*n*		59	25	21	9	3	1
Age group, (y)	0–9	10 (17)	8 (32)	2 (10)	0 (0)	0 (0)	0 (0)
	10–17	4 (7)	2 (8)	2 (10)	0 (0)	0 (0)	0 (0)
	18–39	22 (37)	12 (48)	3 (14)	5 (50)	2 (67)	0 (0)
	40+	23 (39)	3 (12)	14 (67)	4 (50)	1 (33)	1 (100)
Age, (y)		33 [20,48]	23 [7,33]	47 [31,63]	38 [30,48]	39 [38,47]	70
Gender	Male	27 (46)	12 (48)	8 (38)	3 (33)	3 (100)	1 (100)
Ethnicity	White	49 (83)	18 (72)	19 (90)	8 (89)	3 (100)	1 (100)
	Asian	7 (12)	5 (20)	1 (5)	1 (11)	0 (0)	0 (0)
	Mixed	2 (3)	2 (8)	0 (0)	0 (0)	0 (0)	0 (0)
	Other	1 (2)	0 (0)	1 (5)	0 (0)	0 (0)	0 (0)
Participant employment	Paid (FT/PT)	17 (29)	3 (12)	8 (38)	5 (56)	1 (33)	0 (0)
	Unemployed	17 (29)	8 (32)	3 (14)	4 (44)	2 (67)	0 (0)
	Retired	7 (12)	0 (0)	6 (29)	0 (0)	0 (0)	1 (100)
	N/A (child/HE)	18 (31)	14 (56)	4 (19)	0 (0)	0 (0)	0 (0)
Paid (FT/PT) parent employment		14 (24)	10 (40)	4 (19)	0 (0)	0 (0)	0 (0)
Social situation	Independent	35 (59)	6 (24)	16 (76)	9 (100)	3 (100)	1 (100)
	Lives with parent	24 (41)	19 (76)	5 (24)	0 (0)	0 (0)	0 (0)
Number of reviews, n		6 [4,7]	7 [5,8]	5 [3,6]	7 [6,7]	5 [4,5]	5
Period of reviews (years)		6 [4,6]	6 [4,6]	6 [2,6]	6 [6,6]	6 [4,6]	4
BEBS score (clinician)^a^		27 [11,37]	37 [32,45]	14 [5,22]	12 [8,14]	30 [26,35]	7
iscorEB clinician score^b^		21 [7,31] (*n* = 50)	31 [24,37] (*n* = 21)	10 [6,21] (*n* = 17)	6 [5,7]	26 [22,31]	
iscorEB self‐reported severity^b^		44 [26,57] (*n* = 58)	50 [39,59] (*n* = 24)	25 [11,49]	34 [26,54]	60 [57,66]	52
iscorEB skin wounding area score^c^		9 [2,15] (*n* = 54)	14 [12,20] (*n* = 22)	2 [0,6] (*n* = 19)	2 [0,3]	15 [12,20]	2
BEBS %surface area affected^d^		7 [2,15]	15 [8,22]	2 [1,5]	1 [0,1]	22 [15,24]	2
Who does care?							
Self‐caring		16 (27)	0 (0)	12 (57)	2 (22)	1 (33)	1 (100)
Self plus carer		10 (17)	5 (20)	3 (14)	2 (22)	0 (0)	0 (0)
Carer		26 (44)	20 (80)	4 (19)	0 (0)	2 (67)	0 (0)
None required		7 (12)	0 (0)	2 (10)	5 (56)	0 (0)	0 (0)
Dressing frequency							
Changed all at one time		46 (78)	20 (80)	18 (86)	4 (44)	3 (100)	1 (100)
Changed a few at a time		5 (8)	5 (20)	0	0	0	0 (0)
No dressings required		7 (12)	0	2 (10)	5 (56)	0	0 (0)
Infrequent dressing changes		1 (2)	0	1 (5)	0	0	0 (0)
Weekly dressing changes (hours)		7 [1,17]	18 [12,21]	2 [1,4]	0 [0,2]	10 [10,26]	3

*Note*: Results are presented as median [IQR] or *n* (%) for RDEB severe (RDEB‐S), intermediate (RDEB‐I), inversa (RDEB‐Inv), pretibial (RDEB‐PT), pruriginosa (RDEB‐Pru). See Table S1 for participant severity reported as average of all 330 reviews.

Abbreviations: FT/PT, full‐time/part‐time; HE, higher education, for adults; N/A, not applicable.

^a^ Birmingham Epidermolysis Bullosa Severity (BEBS) score, maximum of 100 with higher score indicating greater severity.

^b^ Instrument for scoring clinical outcomes of research for epidermolysis bullosa (iscorEB), maximum score is 138 for clinician score and 120 for self‐reported score, with higher score indicating greater severity.

^c^ iscorEB skin wounding area score (maximum of 60, a component of iscorEB clinician score) includes intact skin, erosions, crusting/scabbing, chronic wounds (>6 weeks), infection and percentage body surface area affected.

^d^ BEBS body surface area affected, a component of BEBS, reports percentage damaged skin including blisters, erosions, healing skin, erythema and atrophic scarring.

### 4.1 Total cost of community care

The median annual per person cost of all RDEB community care at index review was £14 124 [1722; 87 937], including all wound care materials, skin care, paid and proportionate unpaid care, and EB‐CNS home visits (Table 2). Substantially higher costs were reported by individuals with RDEB‐S and RDEB‐Pru, respectively, £89 988 [26 657; 130 115] and £44 880 [69 064;353 454], with the least cost in RDEB‐I, £1906 [782; 7372]. Similar average costs were found when all reviews (*n* = 330) were considered, £16 863 [2699; 93 989] (Table S2), and a Spearman's rank correlation revealed higher overall costs were strongly associated with worse BEBS severity scores (0.73 [0.67;0.78], *n* = 301). However, correlations between overall costs and BEBS severity scores revealed a moderate relationship for RDEB‐I (0.59 [0.43;0.71] (*n* = 88)) and RDEB‐Inv (0.43 [0.18;0.63] (*n* = 51)), but only a weak relationship for RDEB‐S (0.34 [0.18;0.47] (*n* = 145)). This disparity in the total and subgroup correlations is due to the subgroups having very different distributions for cost and severity score.

**TABLE 2:** Annual participant treatment costs (GBP) in 1000s at index review by recessive dystrophic epidermolysis bullosa (RDEB) subtype (*n* = 59).

Characteristic	Overall	RDEB‐S	RDEB‐I	RDEB‐Inv	RDEB‐Pru
Wound dressings	12 [2,75]	77 [22,104]	2 [0,6]	0 [0,10]	33 [32,310]
Tubular bandages	0.1 [0.0,0.8]	0.5 [0.2,1.3]	0.0 [0.0,0.1]	0.0 [0.0,0.0]	2.9 [1.5,4.2]
Retention garments	0.0 [0.0,0.6]	0.0 [0.0,1.7]	0.0 [0.0,0.2]	0.0 [0.0,0.0]	0.0 [0.0,2.7]
Skin care products	0.1 [0,0.3]	0.2 [0.0,0.6]	0.0 [0.0,0.1]	0.0 [0.0,0.0]	0.6 [0.3,1.0]
**Total cost of wound & skin care products**	**12 [2,76]**	**78 [22,106]**	**2 [1,7]**	**0 [0,10]**	**37 [35,313]**
Paid care	0 [0,0]	0 [0,8]	0 [0,0]	0 [0,0]	0 [0,36]
Unpaid care	0 [0,0]	0 [0,11]	0 [0,0]	0 [0,0]	0 [0,3]
**Total paid and unpaid care**	**0 [0,11]**	**11 [3,17]**	**0 [0,0]**	**0 [0,0]**	**6 [3,40]**
EB‐CNS care	0.2 [0.0,0.5]	0.5 [0.0,1.0]	0.0 [0.0,0.5]	0.0 [0.0,0.5]	0.5 [0.2,0.7]
**Total annual cost of all RDEB care**	14 [2,88] (*n* = 59)	90 [27 130] (*n* = 25)	2 [1,7] (*n* = 21)	1 [0,12] (*n* = 9)	45 [39,353] (*n* = 3)

*Note*: Results are presented as median [IQR]. Prices in British Pounds (GBP) as at August 2020. See Table S2 for annual participant treatment costs reported as average of all 330 reviews. The bold values indicate sum of rows above, i.e. 'Total cost of wound & skin care products' refers to the 4 rows above; 'total paid & unpaid care' refers to the 2 rows above; and 'Total annual cost of all RDEB care' refers to the sum of the 7 items in the non‐bold rows.

**FIGURE 1:**
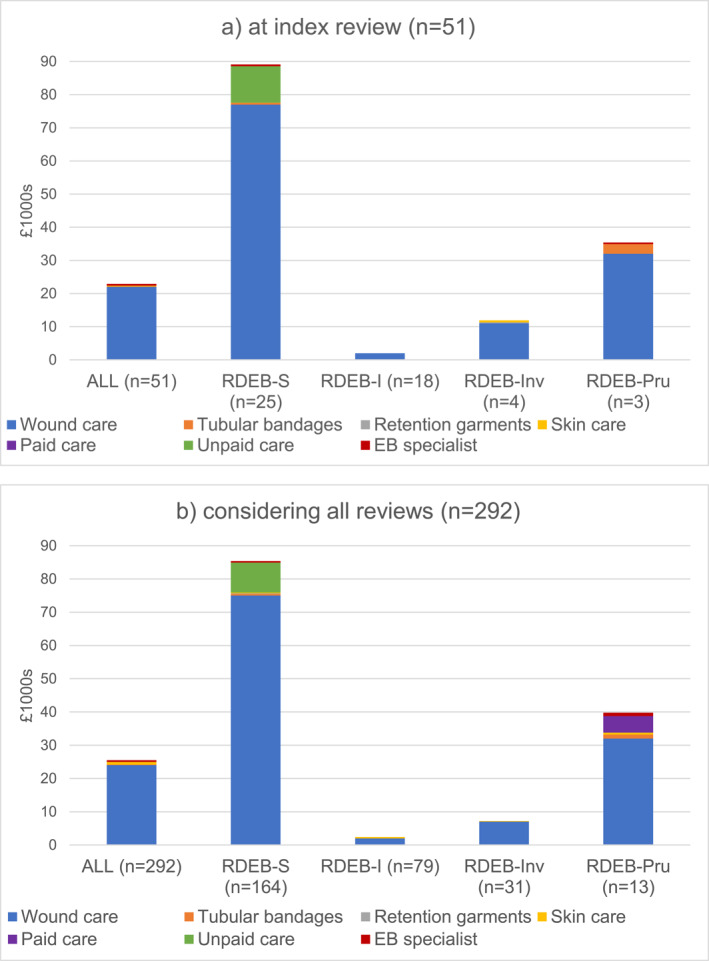
(a) Median annual costs for those reporting frequent dressing changes at index review (*n* = 51). RDEB severe (RDEB‐S), intermediate (RDEB‐I), inversa (RDEB‐Inv), pretibial (RDEB‐PT), pruriginosa (RDEB‐Pru). Prices as at August 2020. RDEB‐PT (*n* = 1) is included in RDEB‐ALL costs only. See Table S3 for dataset used in Figure 1a. (b) Median annual costs for those reporting frequent dressing changes, all reviews (*n* = 292). Prices as at August 2020. RDEB‐PT (*n* = 1) is included in RDEB‐ALL costs only. See Table S4 for dataset used in Figure 1b.

The median annual per person cost for those reporting regular dressing changes at index review (*n* = 51) increased to £26 657 [6019;98 226] after excluding reviews reporting infrequent/no dressing changes (Figure 1a). Consideration of all reviews reporting regular dressing changes (*n* = 292) suggests the annual cost per person could be higher at £33 628 [5738;101 271] (Figure 1b). Data for Figures 1a and 1b are reported in Tables S3 and S4, respectively.

There were minimal or no community care costs for reviews where participants with RDEB‐I or RDEB‐Inv reported infrequent/no dressing changes, although some reported occasional small use of wound care materials. These participants had £0 [0;0] costs at index review, whereas, when considering all reviews, the annual costs were £107 [48;571] for those reporting infrequent dressing changes (*n* = 13) and just £14 [0;67] for those reporting no dressings (*n* = 25).

#### 4.1.1 Cost of wound care products

Table 3 shows the variation in use of wound care products, reporting the number using each treatment component and user median annual costs at index review (*n* = 59). Some participants used multiple care components whereas others reported only one or two products. Individuals with RDEB‐S and RDEB‐Pru reported the greatest wound dressing costs, £77 154 [21 891; 104 140] and £32 686 [32 322; 310 005], respectively. However, it should be noted that 1 of the 3 participants with RDEB‐Pru had unusually high dressing materials costs due to extensive wounding, a large body size, and a personal preference for frequent changes and applying dressings to protect intact skin. This did not affect median costs at index review but is reflected in the IQR. Individuals with RDEB‐I reported little usage and low costs, with 5 of the 9 individuals with RDEB‐Inv reporting no wound dressings. Similar usage and annual costs were found when considering all available reviews (*n* = 330) (Table S5).

**TABLE 3:** Annual usage of wound care products, paid and unpaid care, and EB‐CNS support at index review (*n* = 59), with median user costs for each item reported in 1000s (GBP).

Characteristic	Overall	RDEB‐S	RDEB‐I	RDEB‐Inv	RDEB‐Pru
All participants	59	25	21	9	3
Number (%) with frequent wound dressing changes	51 (86)	25 (100%)	18 (86%)	4 (44%)	3 (100%)
User cost: Wound dressings	22 [5,86]	77 [22,104]	2 [1,7]	11 [9,12]	32 [32,310]
Number (%) using tubular bandages	33 (56)	21 (84)	7 (33)	1 (11)	3 (100)
User cost: tubular bandage	0.6 [0.2,1.3]	0.9 [0.3,1.5]	0.2 [0.1,0.5]	1.2 [1.2,1.2]	2.9 [1.5,4.2]
Number (%) using retention garments	22 (37)	12 (48)	7 (33)	2 (22)	1 (33)
User cost: retention garments	1.0 [0.5,2.4]	1.7 [0.6,2.4]	0.4 [0.2,1.8]	0.7 [0.6,0.7]	5.4 [5.4,5.4]
Number (%) using skin care products	36 (61)	19 (76)	11 (52)	3 (33)	3 (100)
User cost: skin care products	0.2 [0.1,0.6]	0.3 [0.2,0.7]	0.1 [0.1,0.5]	0.1 [0.0,0.2]	0.6 [0.3,1.0]
Number (%) using paid carers	13 (22)	10 (40)	1 (5)	1 (11)	1 (33)
User cost: Paid care	10 [8,28]	14 [6,26]	10 [10,10]	9 [9,9]	73 [73,73]
Number (%) using unpaid carers^a^	14 (24)	10 (40)	3 (14)	0	1 (33)
User cost: Unpaid care	12 [7,16]	12 [11,16]	2 [1,12]		6 [6,6]
Number (%) using EB‐CNS care	29 (49)	17 (68)	7 (33)	3 (33)	2 (67)
User cost: EB‐CNS care	0.5 [0.5,1.0]	1.0 [0.5,1.0]	0.5 [0.5,0.5]	1.0 [0.7,1.2]	0.7 [0.6,0.8]

*Note*: Results are presented as *n* (%) and median [IQR]. Prices as at August 2020. NB: this table reports median per user costs whereas Table 2 reports median costs for all participants. See Table S5 for annual per user costs reported as median for all 330 reviews.

Abbreviation: EB‐CNS, Clinical Nurse Specialist supporting individuals with epidermolysis bullosa.

^a^ Costs for only those reporting care by unpaid carers; excluded those reporting paid + unpaid care and unpaid + self care as not possible to calculate hours of unpaid care for these.

**FIGURE 2:**
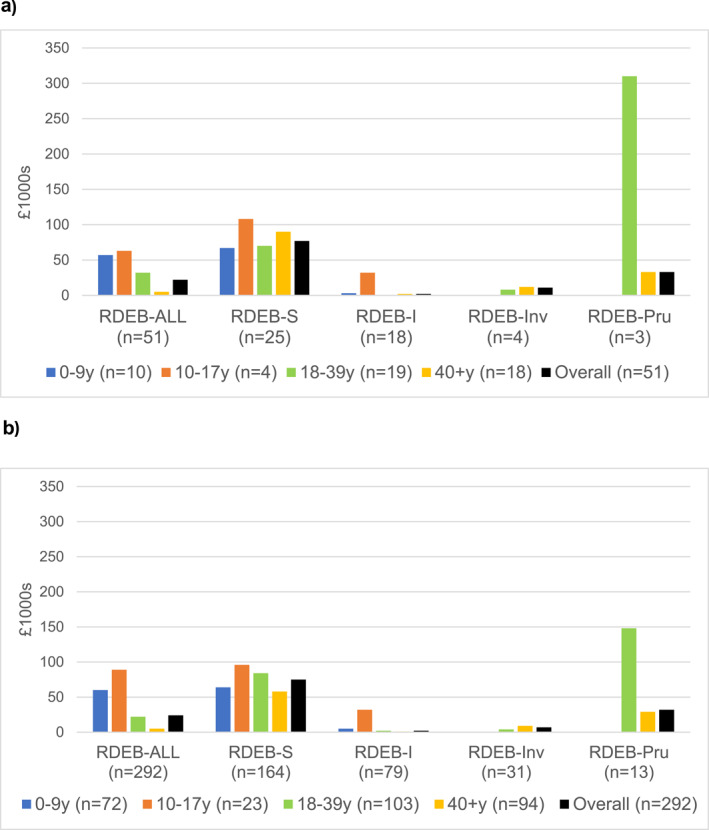
(a) Annual wound dressing costs reported at index review for regular users (*n* = 51) by subtype and age group, per 1000s GBP.Subtypes RDEB = Inv and RDEB = Pru did not include children. RDEB‐PT (*n* = 1) is included in RDEB = ALL costs only. See Table S6 for dataset used in Figure 2. Prices as at August 2020. (b) Annual wound dressing costs reported for regular users by subtype and age group, per 1000s GBP, considering all reviews (*n* = 292). Subtypes RDEB‐Inv and RDEB‐Pru did not include children. RDEB‐PT (*n* = 1) is included in RDEB‐ALL costs only. See Table S6 for dataset used in Figure 2.

Costs were generally lower in young children where wound sizes were proportionally smaller requiring fewer items per product. Figures 2a and 2b show these median costs by age group for each subtype, with data reported in Table S6.

#### 4.1.2 Cost of carers

Only 13 participants reported paid care, with a median 15 [13;43] hours per week at index review and annual user costs of £9750 [8450; 27 950] (Table 3). The overall annual user cost for paid care was higher when considering all reviews, £18 200 [5200; 31 200], with variation between subtype; individuals with RDEB‐S and RDEB‐Inv had higher user costs than reported at index review whereas those with RDEB‐I and RDEB‐Pru had lower user costs (see Table S5).

Paid care was mostly reported by individuals with RDEB‐S and RDEB‐Pru. A single user with RDEB‐Pru reported the greatest usage (112 h per week), with extensive wounding requiring complex and time‐consuming daily dressing changes by 2 carers; this was considered atypical as the other 2 participants with RDEB‐Pru were self‐caring although one received some unpaid care. There were insufficient data to determine whether paid care increased with age.

Most participants (*n* = 46,78%) did not use paid care, although half (*n* = 31,53%) received unpaid care for wound dressing changes, of whom 9 were partly self‐caring and 8 also received paid care. Proportionate annual unpaid carer costs were calculated for the 14 participants (24%) reporting only unpaid carers at index review, a median £11 565 [7394; 15 776] per person (Table 3), with similar proportionate annual unpaid carer costs found when considering all reviews (Table S5).

#### 4.1.3 Cost of specialist nursing care

Most individuals with RDEB‐S (*n* = 17,68%) and RDEB‐Pru (*n* = 2,67%) received a home visit from an EB‐CNS in the 12‐month prior to their index review, including 14 adults (93%) with RDEB‐S, compared with 33% of those with RDEB‐I and RDEB‐Inv. The same pattern was evident when all reviews were considered.

### 4.2 Costs for children with recessive dystrophic epidermolysis bullosa

There were insufficient data to compare costs of care for children with RDEB‐S and RDEB‐I, or child versus adult care costs (Figures 2a and b). However, the trend was for costs to be higher for older children and adults, presumably due to their greater size.

### 4.3 Impact on patient and family

Of the 43 adults who completed QOLEB at index review, 20 (47%) reported their finances (question 3) were greatly or severely affected by RDEB, with a similar percentage affected (*n* = 96,43%) when considering all adult reviews (Table 4). A greater number with RDEB‐S (*n* = 12,80%) reported great or severe impact compared with other subtypes (*n* = 8,30%), whereas nearly half (47%) of those with RDEB‐I reported no financial impact at index review and when considering all reviews. Parents of child‐participants were not asked this question.

**TABLE 4:** Impact of recessive dystrophic epidermolysis bullosa (RDEB) on personal/family finances and carers' ability to work, by subtype at index review (*n* = 59) and all reviews (*n* = 330).

Characteristic		Overall	RDEB‐S	RDEB‐I	RDEB‐Inv	RDEB‐Pru
Adults reporting on financial impact^a^, *n*	Index review	43	15	17	7	3
	All reviews	225	75	85	49	11
No financial impact	Index review	10 (23)	0 (0)	8 (47)	1 (14)	0 (0)
	All reviews	60 (27)	4 (5)	40 (47)	11 (22)	0 (0)
Slightly affected	Index review	13 (30)	3 (20)	4 (24)	4 (57)	2 (67)
	All reviews	69 (31)	21 (28)	17 (20)	26 (53)	5 (45)
Greatly affected	Index review	10 (23)	6 (40)	3 (18)	1 (14)	0 (0)
	All reviews	49 (22)	23 (31)	15 (18)	6 (12)	5 (45)
Severely affected	Index review	10 (23)	6 (40)	2 (12)	1 (14)	1 (33)
	All reviews	47 (21)	27 (36)	13 (15)	6 (12)	1 (9)
Participants^b^, *n*		59	25	21	9	3
Carer unable to work	Index review	21 (36)	14 (56)	5 (24)	1 (11)	1 (33)
	All reviews	105 (32)	84 (51)	14 (15)	4 (8)	3 (23)
Carer reduced work hours	Index review	2 (3)	1 (4)	1 (5)	0	0
	All reviews	18 (5)	15 (5)	3 (3)	0	0
No impact on ability to work	Index review	36 (61)	10 (40)	15 (71)	8 (89)	2 (67)
	All reviews	186 (61)	60 (38)	68 (81)	43 (91)	10 (77)

Results are presented as *n* (%).

^a^ Reporting the number of participants responding to QOLEB question 15: “How are you or your family affected financially by your EB?”

^b^Number of Prospective Epidermolysis Bullosa Longitudinal Evaluation Study (PEBLES) participants in this dataset.

Since the aim of this study was to estimate costs to the NHS, we did not measure costs to patients, families and wider society (such as lost productivity). However, the wider impact of RDEB is indicated through the number of unemployed participants (29%) at index review (Table 1), a median 10 [3;18] hours per week spent changing dressings (Table 1, Table S1), and the need for paid and unpaid care as reported above. Also, 21 participants (36%) reported their carer was unable to work due to the need to provide EB‐related care, and another 2 carers had reduced their hours (Table 4).

## 5 DISCUSSION

This study recorded previously unreported and detailed data relating to costs of community care in individuals with different RDEB subtypes, including costs of dressings and other skincare materials. This paper highlights the high healthcare costs associated with managing wound care for individuals with RDEB‐S and RDEB‐Pru with low costs for RDEB‐I and RDEB‐Inv, and the impact on employment opportunities for patients and their unpaid carers.

Epidermolysis bullosa has a high community and public health cost, with frequent dressing changes driving up the cost of care. The impact of disease severity is evident in differences between median cost of community care for all 59 participants at index review (£14 124) and for those (*n* = 51) requiring regular dressing changes (£26 657). Participants across all subtypes generally used similar wound and skin care materials, so cost variations were largely due to product quantity, relating to disease severity, rather than use of cheaper or more expensive alternatives. The much lower costs for RDEB‐I and RDEB‐Inv groups underscores the need to undertake analysis by subtype in future studies of EB care costs since wound management and care needs can vary significantly. We cannot compare our participants' disease severity with other studies reporting costs as they used different metrics and reporting methods.

Our study found costs were significantly higher in RDEB‐S, reflecting greater symptom severity; of their annual £90 000 costs, £78 234 [22 124; 105 996] were associated with wound and skin care, predominantly dressings. Median total costs for RDEB‐Pru, £44 880, were second greatest, although very high dressing and carer costs in one participant is likely unrepresentative. Markers of severity were highest in the RDEB‐S and RDEB‐Pru groups (albeit only 3 participants had RDEB‐Pru) and these had frequent dressing changes taking a median of 18 and 10 h per week, respectively. In contrast, RDEB‐I and RDEB‐Inv participants had less severe disease, some had infrequent or no dressing changes; those that did need dressings took a median of just 2 and 3 h per week, respectively. Although self‐reported disease severity in EB has been linked to increased care needs and time taken to do dressings,^30^ this has not been formally reported previously nor by RDEB subtype. For comparison, costs of biologic or small molecule drugs for treating adults with common inflammatory diseases such as eczema or psoriasis are currently in the order of £10 000 to £30 000 per annum.^31^

For RDEB‐S, dressing costs were slightly lower for the first decade of life compared to later, which might be expected with more dressings required to cover larger bodies^32^ and chronic wounds becoming more problematic with increasing age.^33^ Dressing costs for the other subtypes are difficult to interpret by age as the number of children with RDEB‐I was low and the RDEB‐Inv and RDEB‐Pru phenotypes tend to manifest in later childhood or adulthood.

The model of care in England and Wales includes centralised EB reference centres with provision of home visits by EB‐CNSs. All dressings and wound care products are provided on prescription and are therefore not a cost to the patient or family. Almost half the participants in our study received at least one EB‐CNS visit in the year prior to their index review; a greater percentage of those with RDEB‐S received visits reflecting their greater clinical need.

We found significant costs for paid and unpaid care, reported by 22% and 24% of our cohort, respectively, and limited to wound changing dressings, with a median cost of £10K and £12K per annum. Most RDEB‐S participants (80%) used paid care, unpaid care, or both at a combined annual median cost of around £26 000. The European study^9^ reported higher costs for both paid and unpaid care when considering DEB in all five countries and for UK, respectively €581 and €2323 for paid carers and €29045 and €21246 for unpaid carers, but asked unpaid carers about all time spent on caring duties, including assisting with activities of daily living; we only reported unpaid carer time for dressing changes so our unpaid care costs are partial in comparison.

The indirect impact of RDEB on ability to work was notable in our study although not quantifiable in financial terms. Firstly, 39% of carers reported inability to work or had reduced work hours due to their caring role, which increased to 60% in RDEB‐S. Secondly, an overall median time of 10 h per week for dressing changes likely contributed to the 50% unemployment rate in employment‐aged adults. Individuals with RDEB‐S reported 73% unemployment and 18 h per week doing dressing changes, although we did not address other medical reasons which might preclude employment. In contrast, Angelis et al. reported a small overall productivity loss of 0.5%,^9^ which suggests their cohort experienced much less impact from EB on employment; this study included dominant DEB which is frequently milder than RDEB with less impact on ability to work.

Almost half the adult participants reported great or severe impact to their finances from their EB, rising to 80% for RDEB‐S, although almost half of those with RDEB‐I felt no EB‐related financial impact. This further highlights the need for subtype analysis when assessing costs and financial impact in EB. The financial impact for families in other countries where dressings are not fully reimbursed is likely to be significantly greater than for participants in our UK study who received all dressing materials through the NHS with costs fully covered apart from a small prescription charge for eligible adults.

The variation in data collection methods and models for funding healthcare makes any meaningful comparisons between study findings difficult. A European multinational questionnaire‐based study demonstrated significantly higher average annual costs for DEB (€53,359) compared with all forms of EB (€31,390) but did not separately report costs for RDEB.^8^ ^,^ ^9^ Also, that study did not include costs of dressing materials although reported direct medical costs (e.g. hospital admissions and appointments) and indirect costs, including lost income from being unable to work, which we did not. A small Irish study reported combined costs of wound care materials and medications for 4 children with RDEB (from €5986 to €89,780), and hospital admissions and clinic visits, but did not include paid and unpaid carer time.^27^ A recent 2022 study from the United States used data from health records and insurance claims for medical and home care (including wound dressing materials, pharmacy) for 26 patients with RDEB (subtypes unspecified) and calculated average annual care costs of $29,995^28^; however, although bandage costs ($5341) and home nursing and wound care materials ($7615) were included, the cost of wound care materials not reimbursed by their insurers is unknown so it is not possible to determine the total cost of paid care or wound dressings. A separate United States online survey of EB patients and their carers found that more than 25% spent over $1000 per month on wound care supplies to supplement those covered by health insurance and almost 75% of families with severe EB experienced a major or moderate financial impact.^29^

A strength of our study is the inclusion of RDEB subtypes, adults and children, who have contributed many reviews over several years. Limitations which can make interpretation problematic include the small sample size and underrepresentation of paediatric participants and those with less common RDEB subtypes, including skewing of RDEB‐Pru data by one participant; this should improve with recruitment of additional PEBLES participants. Our results assume steady use of dressings and care over the preceding 6 or 12 months since previous review such that fluctuations in weather or other factors do not influence costs incurred in care. Also, our findings underrepresent informal care and do not include other relevant healthcare costs (hospital care, investigations, medicines).

## 6 CONCLUSIONS

This study demonstrates the very considerable costs of community care for RDEB, underscoring the particularly high costs for more severe forms of RDEB, notably RDEB‐S. It comprehensively details community care costs in different RDEB subtypes, including paid and proportionate unpaid carer costs, and highlights the many hours per week spent undertaking dressing changes which contributes to the considerable burden and financial impact reported by affected families, including reduced ability of carers and individuals with EB to undertake paid work. This information supports the need for individuals with EB and their carers to have adequate provision of community care, including reimbursement of dressing materials and skincare products. The study findings also support the health economic case for developing new therapies for RDEB which might accelerate wound healing, diminish total wound burden, and thereby reduce costs of dressings and care.

## CONFLICT OF INTEREST STATEMENT

AEM and JEM have undertaken paid consultancy for Amryt Pharma and Krytsal Biotech.

## AUTHOR CONTRIBUTIONS

**Eunice Jeffs**: Conceptualization (equal); Data curation (equal); Formal analysis (supporting); Funding acquisition (supporting); Investigation (equal); Methodology (equal); Project administration (lead); Resources (equal); Writing – original draft (lead); Writing – review & editing (equal). **Elizabeth Pillay**: Conceptualization (equal); Funding acquisition (supporting); Investigation (equal); Methodology (equal); Resources (equal); Writing – original draft (supporting); Writing – review & editing (equal). **Lesedi Ledwaba‐Chapman**: Data curation (equal); Formal analysis (lead); Writing – review & editing (equal). **Alessandra Bisquera**: Formal analysis (supporting); Writing – review & editing (equal). **Susan Robertson**: Writing – review & editing (equal). **John McGrath**: Writing – review & editing (equal). **Yanzhong Wang**: Formal analysis (supporting); Writing – review & editing (equal). **Anna Martinez**: Writing – review & editing (equal). **Anita Patel**: Conceptualization (supporting); Formal analysis (supporting); Writing – review & editing (equal). **Jemima Mellerio**: Conceptualization (equal); Funding acquisition (lead); Methodology (equal); Supervision (lead); Writing – original draft (supporting); Writing – review & editing (equal).

## ETHICS STATEMENT

Prospective Epidermolysis Bullosa Longitudinal Evaluation Study was ethically approved by the UK NRES Committee London‐Bromley (REC 15/LO/001) and Health Research Authority (IRAS 142032). All subjects provided written informed consent to participate in the study and participated voluntarily with no incentives offered, financial or otherwise. The study has been conducted in accordance with the ethical principles in the Declaration of Helsinki and consistent with Good Clinical Practice and applicable UK regulatory requirements.

## Supporting information

Supplementary Material

## Data Availability

The data underlying this article will be shared on reasonable request to the corresponding author.
