# Supplementary material for: Costs of UK community care for individuals with recessive dystrophic epidermolysis bullosa: Findings of the Prospective Epidermolysis Bullosa Longitudinal Evaluation Study
Source: Skin Health Dis. 2024 Jan 10;4(1):e314. doi: 10.1002/ski2.314 (PMC10831550; doi:10.1002/ski2.314)
Supplement: Supplementary file 1 — Supplementary Material [file SKI2-4-e314-s001.docx]

# **Supplementary file for review**

## **Appendix S1: Examples of PEBLES questions**

**Skin care/wound care regime**

- Frequency of dressings (tick one, the most common frequency): □all at once; □few at a time; □infrequent; □none needed
- Dressings undertaken by (tick all that apply): □self; □parent; □paid carer; □informal/unpaid carer
- Number of dressing changes per week __________
- Number of patch-ups per week ___________
- Average preparation time for each dressing change (mins): ____________
- Average time taken to change dressings (mins): _______________
- Average time taken to do patch-ups (mins): _____________

**Wound care products**

Dressings: Please list all dressings used (including flat bandages, e.g. K band): name, size, quantity used in a week, e.g. Mepilex Transfer, 20x50cm, 3 pieces

Retention garments: list Skinnies WEB and/or Skinnies/Comfifast garments used over 6-months, e,g, Skinnies ‘WEB’ full 5 digit gloves, size 5, 2 pairs

Tubular bandages: list quantity used in a week, e.g. Tubifast 7.5cm x 3m, 1m

**Skin care products**

Please complete for items used in a week.

Ointments/creams: e.g. Epaderm ointment, 30g, used 33%/10g

Bath/shower additives or soap substitutes: e.g. Hydromol bath and shower emollient, 350ml, 100%/350ml

**Funding of care**

- How is care funded (tick all that apply): □not funded; □direct payments; □continuing care; □NHS; □privately funded
- Number of paid hours care per week: Band 1-8a or hourly rate of pay, hours worked, e.g. 5 hours at Band 6, or rate of pay per hour
- Number of **unpaid** hours per week: parent carer: ______; other carer _____

**Social cost of care**

- Does caring for individual with EB mean carer unable to seek paid employment? □yes; □no; □not applicable as self-caring
- Has the carer had to give up paid work or reduce work hours to care for the person with EB? □yes; □no; □not applicable as self-caring
- Does the parent-carer receive carer’s allowance? □yes; □no

## **Table S1: Participant severity by recessive dystrophic epidermolysis bullosa (RDEB) subtype, considering all reviews (n=330)**

| Characteristic | Overall^1^ | RDEB-S | RDEB-I | RDEB-Inv | RDEB-Pru |
| --- | --- | --- | --- | --- | --- |
| n | 330 | 164 | 95 | 53 | 13 |
| BEBS score (clinician)^2^ | 24 [12,37] (n=301) | 36 [27,44] (n=145) | 13 [6,24] (n=88) | 12 [8,16] (n=51) | 29 [23,37] (n=12) |
| iscorEB clinician score^3^ | 21 [10,32] (n=234) | 29 [21,39] (n=121) | 10 [7,21] (n=62) | 6 [5,11] (n=39) | 25 [18,28] (n=11) |
| iscorEB self-reported severity^3^ | 42 [26,56]  (n = 313) | 46 [36,56]  (n = 152) | 26 [11,52] (n=93) | 36 [26,52] (n=52) | 70 [60,75] (n=11) |
| iscorEB skin wounding area score^4^ | 8 [2,15] (n=218) | 14 [12,21]  (n = 104) | 2 [0,5]  (n = 64) | 2 [0,4] (n=39) | 13 [8,18] (n=8) |
| BEBS body surface area affected^5^, % | 8 [2,15] (n=302) | 14 [9,20] (n=146) | 2 [0,6] (n=88) | 1 [0,4] (n=51) | 19 [14,28] (n=12) |
| *Dressing frequency* |  |  |  |  |  |
| - Changed all at one time | 246 (75) | 129 (79) | 71 (75) | 28 (53) | 13 (100) |
| - Changed a few at a time | 46 (14) | 35 (21) | 8 (8) | 3 (6) | 0 |
| - No dressings required | 25 (8) | 0 | 8 (8) | 17 (32) | 0 |
| - Infrequent dressing changes | 13 (4) | 0 | 8 (8) | 5 (9) | 0 |
| Weekly dressing changes (hours) | 7 [1,17] | 16 [9,23] | 1 [0,4] | 0 [0,3] | 21 [10,36] |
| *Who does care?* |  |  |  |  |  |
| - Self-caring | 95 (29) | 6 (4) | 61 (64) | 24 (46) | 1 (8) |
| - Self plus carer | 44 (13) | 16 (10) | 15 (16) | 8 (15) | 3 (23) |
| - Carer | 165 (50) | 142 (87) | 11 (12) | 3 (6) | 9 (69) |
| - None required | 25 (8) | 0 (0) | 8 (8) | 17 (32) | 0 (0) |
| Correlation between BEBS score and annual cost of care^6^ | 0.73 [0.67,0.78] (n = 301) | 0.34 [0.18,0.47] (n = 145) | 0.59 [0.43,0.71] (n = 88) | 0.43 [0.18,0.63] (n = 51) | 0.44 [-0.18,0.81] (n = 12) |

*Results are presented as median [IQR] or n (%). RDEB severe (RDEB-S), intermediate (RDEB-I), inversa (RDEB-Inv), pretibial (RDEB-PT), pruriginosa (RDEB-Pru)*

*^1^ Five reviews by one individual with RDEB-PT were included in overall analysis but not separately reported*

*^2^ Birmingham Epidermolysis Bullosa Severity (BEBS) score, maximum of 100 with higher score indicating greater severity.*

*^3^ Instrument for scoring clinical outcomes of research for epidermolysis bullosa (iscorEB), maximum score is 138 for clinician score and 120 for self-reported score, with higher score indicating greater severity.*

*^4^ iscorEB skin wounding area score (maximum of 60, a component of iscorEB clinician score) includes intact skin, erosions, crusting/scabbing, chronic wounds (>6 weeks), infection and percentage body surface area*

*^5^ BEBS body surface area affected is one component of BEBS, reporting percentage damaged skin including blisters, erosions, healing skin, erythema and atrophic scarring.*

## **Table S2: Annual participant treatment costs (GBP) in 1000s by RDEB subtype, reporting average of all reviews (n=330)**

| Characteristic | Overall^1^ | RDEB-S | RDEB-I | RDEB-Inv | RDEB-Pru |
| --- | --- | --- | --- | --- | --- |
| Wound dressings | 10 [1,80] | 75 [23,112] | 1 [1,6] | 1 [0,8] | 32 [20,264] |
| Tubular bandages | 0.1 [0.0,0.7] | 0.5 [0.1,1.1] | 0.0 [0.0,0.1] | 0.0 [0.0,0.0] | 1.2 [0.3,1.9] |
| Retention garments | 0.0 [0.0,0.6] | 0.2 [0.0,1.5] | 0.0 [0.0,0.0] | 0.0 [0.0,0.0] | 0.0 [0.0,0.9] |
| Skin care products | 0.1 [0.0,0.3] | 0.2 [0.0,0.6] | 0.0 [0.0,0.1] | 0.0 [0.0,0.1] | 0.6 [0.1,1.4] |
| **Total cost of wound & skin care products** | **10 [2,82]** | **77 [24,114]** | **1 [1,6]** | **2 [0,8]** | **33 [22,269]** |
| Paid care | 0 [0,2] | 0 [0,14] | 0 [0,0] | 0 [0,0] | 5 [0,48] |
| Unpaid care | 0 [0,1] | 0 [0,10] | 0 [0,0] | 0 [0,0] | 0 [0,0] |
| **Total paid and unpaid care** | **1 [0,12]** | **11 [3,20]** | **0 [0,0]** | **0 [0,0]** | **9 [0,48]** |
| EB-CNS care | 0.5 [0.0,1.0] | 0.5 [0.0,1.0] | 0.0 [0.0,0.5] | 0.0 [0.0,0.5] | 1.0 [0.0,1.0] |
| **Total annual cost of all RDEB care** | 17 [3,94]  (n = 330) | 87 [33,139]  (n=164) | 1 [1,7]  (n = 95) | 4 [0,12]  (n = 53) | 45 [34,311]  (n = 13) |
| Correlation between BEBS score and annual cost of care^2^ | 0.73 [0.67,0.78] (n = 301) | 0.34 [0.18,0.47] (n = 145) | 0.59 [0.43,0.71] (n = 88) | 0.43 [0.18,0.63] (n = 51) | 0.44 [-0.18,0.81] (n = 12) |

*Results are presented as median [IQR]. Costs as at August 2020.*

*^1^Five reviews by one individual with RDEB-PT were included in overall analysis but not separately reported*

*^2^ Results are presented as correlation [95% CI] (n) and calculated using Spearman’s rank correlation. Associations are significant if the 95% CI does not contain 0. Correlations can be interpreted as a negligible relationship (<0.2), weak (0.2-0.4), moderate (0.4-0.6), strong (0.6-0.8), or very strong relationship (>0.8).*

## **Table S3: Data use in Fig 1a, Annual participant treatment costs (GBP) in 1000s at index review by RDEB subtype for those reporting regular dressing changes (n=51)**

| Characteristic | Overall | RDEB-S | RDEB-I | RDEB-Inv | RDEB-Pru |
| --- | --- | --- | --- | --- | --- |
| Number (%) with frequent dressing changes | 51 (86%) | 25 (100%) | 18 (86%) | 4 (44%) | 3 (100%) |
| Wound dressings | 22 [5,86] | 77 [22,104] | 2 [1,7] | 11 [9,12] | 33 [32,310] |
| Tubular bandages | 0.2 [0.0,1.1] | 0.5 [0.2,1.3] | 0.0 [0.0,0.1] | 0.0 [0.0,0.3] | 2.9 [1.5,4.2] |
| Retention garments | 0.0 [0.0,0.6] | 0.0 [0.0,1.7] | 0.0 [0.0,0.3] | 0.3 [0.0,0.6] | 0.0 [0.0,2.7] |
| Skin care products | 0.1 [0.0,0.5] | 0.2 [0.0,0.6] | 0.1 [0.0,0.2] | 0.0 [0.0,0.1] | 0.6 [0.3,1.0] |
| **Total cost of wound & skin care products** | **22 [6,88]** | **78 [22,106]** | **2 [1,8]** | **11 [10,12]** | **37 [35,313]** |
| Paid care | 0 [0,1] | 0 [0,8] | 0 [0,0] | 0 [0,2] | 0 [0,36] |
| Unpaid care^1^ | 0 [0,1] | 0 [0,11] | 0 [0,0] | 0 [0,0] | 0 [0,3] |
| **Total paid and unpaid care^1^** | **2 [0,12]** | **11 [3,17]** | **0 [0,0]** | **0 [0,2]** | **6 [3,40]** |
| EB-CNS care | 0.5 [0.0,0.5] | 0.5 [0.0,1.0] | 0.0 [0.0,0.5] | 0.0 [0.0,0.4] | 0.5 [0.2,0.7] |
| **Total annual cost of all RDEB care** | **27 [6,98]** | **90 [27,130]** | **3 [1,8]** | **13 [11,15]** | **45 [39,353]** |

*Results are presented as number (%) and* *median [IQR]. Costs as at August 2020.*

*^1^Costs for unpaid care are an underestimate as only available for those who did not also receive paid care.*

## **Table S4: Data use in Fig 1b, Annual participant treatment costs (GBP) in 1000s with median user costs for each item reported in 1000s (GBP) as an average of all reviews (n=330), for those 51 participants reporting regular dressing changes**

| Characteristic | Overall | RDEB-S | RDEB-I | RDEB-Inv | RDEB-Pru |
| --- | --- | --- | --- | --- | --- |
| Number (%) with frequent dressing changes | 292 (88%) | 164 (100%) | 79 (83%) | 31 (58%) | 13 (100%) |
| Wound dressings | 24 [4,85] | 75 [23,112] | 2 [1,7] | 7 [4,12] | 32 [20,264] |
| Tubular bandages | 0.2 [0.0,0.8] | 0.5 [0.1,1.1] | 0.0 [0.0,0.2] | 0.0 [0.0,0.0] | 1.2 [0.3,1.9] |
| Retention garments | 0.0 [0.0,0.9] | 0.2 [0.0,1.5] | 0.0 [0.0,0.0] | 0.0 [0.0,0.2] | 0.0 [0.0,0.9] |
| Skin care products | 0.8 [0,0.4] | 0.2 [0,0.6] | 0.4 [0,0.1] | 0.2 [0,0.2] | 0.6 [1.0,1.4] |
| **Total cost of wound & skin care products** | **25 [4,87]** | **77 [24,114]** | **2 [1,7]** | **7 [4,12]** | **33 [22,269]** |
| Paid care | 0 [0,3] | 0 [0,14] | 0 [0,0] | 0 [0,0] | 5 [0,48] |
| Unpaid care^1^ | 0 [0,3] | 0 [0,10] | 0 [0,0] | 0 [0,0] | 0 [0,0] |
| **Total paid and unpaid care^1^** | **3 [0,14]** | **11 [3,20]** | **0 [0,0]** | **0 [0,1]** | **9 [0,48]** |
| EB-CNS care | 0.5 [0.0,1.0] | 0.5 [0.0,1.0] | 0.0 [0.0,0.5] | 0.0 [0.0,1.0] | 1.0 [0.0,1.0] |
| **Total annual cost of all RDEB care** | **34 [6,101]** | **87 [33,139]** | **3 [1,8]** | **9 [5,16]** | **45 [34,311]** |

*Results are presented as number (%) and median [IQR]. Costs as at August 2020.*

*^1^Costs for unpaid care are an underestimate as only available for those who did not also receive paid care.*

## **Table S5: Annual usage of wound care products, paid and unpaid care, and EB-CNS support, with median user costs for each item reported in 1000s (GBP) as an average of all reviews (n=330)**

| Characteristic | Overall | RDEB-S | RDEB-I | RDEB-Inv | RDEB-Pru |
| --- | --- | --- | --- | --- | --- |
| All participants | 330 | 164 | 95 | 53 | 13 |
| Number (%) with frequent wound dressing changes | 292 (88) | 164 (100) | 79 (83) | 31 (58) | 13 (100) |
| User cost: Wound dressings | 24 [4,87] | 75 [23,113] | 2 [1,7] | 7 [4,12] | 33 [22,264] |
| Number (%) using tubular bandages | 180 (55) | 128 (78) | 31 (33) | 4 (8) | 13 (100) |
| User cost: tubular bandage | 0.6 [0.2,1.2] | 0.6 [0.3,1.3] | 0.3 [0.1,0.7] | 1.2 [0.9,1.2] | 1.2 [0.3,1.9] |
| Number (%) using retention garments | 121 (37) | 86 (52) | 20 (21) | 9 (17) | 6 (46) |
| User cost: retention garments | 1.2 [0.5,2.3] | 1.4 [0.6,2.4] | 0.8 [0.2,1.5] | 0.6 [0.5,1.2] | 1.1 [0.8,2.1] |
| Number (%) using skin care products | 205 (62) | 114 (70) | 53 (56) | 26 (49) | 11 (85) |
| User cost: skin care products | 0.2 [0.1,0.6] | 0.4 [0.2,0.7] | 0.1 [0.0,0.2] | 0.1 [0.0,0.3] | 0.6 [0.3,1.9] |
| Number (%) using paid carers | 89 (27) | 69 (42) | 5 (6) | 7 (13) | 7 (54) |
| User cost: Paid care | 18 [5,31] | 19 [7,30] | 3 [2,6] | 7 [4,9] | 48 [25,61] |
| Number (%) using unpaid carers^1^ | 84 (25) | 72 (44) | 2 (2) | 1 (2) | 2 (15) |
| User cost: Unpaid care | 10 [5,16] | 12 [7,16] | 2 [2,3] | 5 [5,5] | 9 [8,10] |
| Number (%) using EB-CNS care | 165 (50) | 112 (68) | 29 (31) | 14 (26) | 9 (69) |
| User cost: EB-CNS care | 1.0 [0.5,1.4] | 1.0 [0.5,1.4] | 0.5 [0.5,1.0] | 1.0 [0.6,1.0] | 1.0 [1.0,1.9] |

*Results are presented as number (%) and median [IQR]. Costs as at August 2020.*

^1^ *Costs for only those reporting care by unpaid carers; excluded those reporting paid+unpaid care and unpaid+self-care as not possible to calculate hours of unpaid care for these*

## **Table S6: Data for Figure 2, Wound dressing costs per 1000s GBP, reported for regular users by subtype and age group, (a) at index review (n=51), (b) for all reviews (n=292)**

| Age group | Overall | RDEB-S | RDEB-I | RDEB-Inv | RDEB-Pru |
| --- | --- | --- | --- | --- | --- |
| **Index reviews** | **22 [6,88]  (n = 51)** | **78 [22,106]  (n = 25)** | **2 [1,8]  (n = 18)** | **11 [10,12]  (n = 4)** | **37 [35,313]  (n = 3)** |
| - 0-9y | 57 [11,77]  (n = 10) | 67 [45,81]  (n = 8) | 4 [3,5]  (n = 2) |  |  |
| - 10-17y | 65 [35,103]  (n = 4) | 112 [103,121]  (n = 2) | 33 [30,35]  (n = 2) |  |  |
| - 18-39y | 37 [7,129]  (n = 19) | 71 [21,165]  (n = 12) | 1 [1,4]  (n = 3) | 9 [8,10]  (n = 2) | 313 [175,451]  (n = 2) |
| - 40+y | 5 [2,27]  (n = 18) | 92 [48,95]  (n = 3) | 2 [1,7]  (n = 11) | 12 [12,12]  (n = 2) | 33 [33,33]  (n = 1) |
| **All reviews** | **25 [4,87]  (n = 292)** | **77 [24,114]  (n = 164)** | **2 [1,7]  (n = 79)** | **7 [4,12]  (n = 31)** | **33 [22,269]  (n = 13)** |
| - 0-9y | 60 [9,86]  (n = 72) | 64 [26,87]  (n = 67) | 6 [4,6]  (n = 5) |  |  |
| - 10-17y | 94 [67,120]  (n = 23) | 97 [79,130]  (n = 21) | 33 [30,35]  (n = 2) |  |  |
| - 18-39y | 22 [5,121]  (n = 103) | 92 [9,146]  (n = 59) | 3 [1,7]  (n = 19) | 6 [2,10]  (n = 17) | 153 [30,502]  (n = 8) |
| - 40+y | 5 [1,24]  (n = 94) | 61 [7,93]  (n = 17) | 2 [1,6]  (n = 53) | 9 [5,13]  (n = 14) | 29 [16,33]  (n = 5) |

*Costs as at August 2020.*
